# Gut microbiome influences incidence and outcomes of breast cancer by regulating levels and activity of steroid hormones in women

**DOI:** 10.1002/cnr2.1847

**Published:** 2023-06-13

**Authors:** Shilpa S. Chapadgaonkar, Srashti S. Bajpai, Mukul S. Godbole

**Affiliations:** ^1^ Department of Biosciences and Technology, Faculty of Sciences and Health Sciences Dr. Vishwanath Karad MIT World Peace University Pune India

**Keywords:** breast cancer, estrogen, gut microbiome, progesterone, therapy response

## Abstract

**Background:**

Breast cancer, the leading cancer type in women worldwide, is affected by reproductive and nonreproductive factors. Estrogen and progesterone influence the incidence and progression of breast cancer. The microbiome of the gut, a complex organ that plays a vital role in digestion and homeostasis, enhances availability of estrogen and progesterone in the host. Thus, an altered gut microbiome may influence the hormone‐induced breast cancer incidence. This review describes the current understanding of the roles of gut microbiome in influencing the incidence and progression of breast cancer, with an emphasis on the microbiome‐induced metabolism of estrogen and progesterone.

**Recent Findings:**

Microbiome has been recognized as a promising hallmark of cancer. Next‐generation sequencing technologies have aided in rapid identification of components of the gut microbiome that are capable of metabolizing estrogen and progesterone. Moreover, studies have indicated a wider role of the gut microbiome in metabolizing chemotherapeutic and hormonal therapy agents and reducing their efficacy in patients with breast cancer, with a predominant effect in postmenopausal women.

**Conclusion:**

The gut microbiome and variations in its composition significantly alter the incidence and therapy outcomes of patients with breast cancer. Thus, a healthy and diverse microbiome is required for better response to anticancer therapies. Finally, the review emphasizes the requirement of studies to elucidate mechanisms that may aid in improving the gut microbiome composition, and hence, survival outcomes of patients with breast cancer.

## 1 INTRODUCTION

The human body hosts trillions of microorganisms, which are located in different organs.^1^ The composition of microbial communities and their genetic contents, together referred to as the microbiome,^2^ varies between individuals. Various groups have used microarray, 16S rRNA sequencing, metagenomic profiling of the entire DNA content, or whole genome shotgun sequencing approaches to examine the microbiome of the human gastrointestinal tract,^1^, ^2^, ^3^, ^4^, ^5^ which has a profound influence on the overall homeostasis and health–disease balance.^6^ For instance, increased levels of *Fusobacterium nucleatum* are associated with the incidence and progression of head and neck cancer.^7^ Specifically, the microbiome of the gut is being considered as an independent organ; it regulates local and systemic processes including, but not limited to, digestion, gut–brain communication, inflammatory responses, and occurrence of diseases.^8^ In general, *Firmicutes*, *Bacteriodetes*, *Fusobacteria*, *Actinobacteria*, *Tenericutes*, *Proteobacteria*, *Verrucomicrobia*, *and Lentisphaerae* are the major phyla; whereas, *Lactobacillus*, *Faecalibacterium*, *Streptococcus*, *Eubacterium*, *Peptococcus*, *Ruminococcus*, *Peptostreptococcus*, and *Bifidobacterium* are the predominant genera in the gut microbiota.^9^, ^10^ Further, gut microbiome controls host nutrition by regulating metabolism of inaccessible dietary products, such as plant polysaccharides, and consumption patterns of animal fats, dietary fibers, and vegetables that are linked to distinct patterns of gut microbiome composition.^11^ The gut microbiome also communicates with microbiomes at other body sites. For instance, the gut microbiome influences the occurrence and progression of skin disorders.^12^, ^13^ Gut dysbiosis—perturbation of the microbiome—increases the risk of developing inflammatory and autoimmune diseases, cancer, obesity, and diabetes.^14^

Breast cancer is the most heterogeneous cancer type, and is the leading cause of cancer‐related deaths in women worldwide. Breast cancer can be broadly categorized into hormone receptor‐positive and ‐negative types.^15^ In addition to the genetic and epigenetic alterations and hereditary factors, the incidence and progression of breast cancer is influenced by several other factors, such as age, duration of exposure to menstruation hormones, breastfeeding, pregnancy, body mass index, and exposure to carcinogens.^16^ For instance, the risk of breast cancer reduces by 4% with a breastfeeding duration of 12 months.^17^

Polymorphic microbiome has recently been recognized as a hallmark of cancer.^18^ Several earlier studies have focused on the role of gut microbiome on the occurrence of gastric and colorectal cancers.^19^ However, recent approaches have observed a close association between gut microbiome and breast cancer.^20^ Specifically, microbial dysbiosis has been found to influence the incidence of different subtypes of breast cancer.^21^, ^22^, ^23^ Furthermore, patients with cancer show more variations in gut microbiota composition than those with benign tumors.^24^ Interestingly, the composition of the gut microbiome also influences patients' response to cancer therapy.^25^ Therefore, the role of the gut microbiome warrants detailed evaluation for understanding the complex interactions with the breast tissue and aid clinicians in improving outcomes of patients with breast cancer.

The levels and duration of exposure to estrogen and progesterone are known risk factors for breast cancer.^26^ Specifically, estrogen and progesterone exert opposing effects, with progesterone conferring a more protective role on the outcomes of breast cancer.^27^ Interestingly, some components of the gut microbiome are capable of metabolizing endogenous and diet‐derived estrogen and progesterone, and thus, either increasing or decreasing their circulating levels.^28^, ^29^ Additionally, menstruation influences the activity of the gut microbiome.^30^ Therefore, this review attempts to discuss the influence of the human gut microbiome on levels and activity of steroid hormones, primarily estrogen, and progesterone, which influence the occurrence and progression of breast cancer.

## 2 INFLUENCE OF MENSTRUAL PHASE ON BREAST CANCER RISK

The breasts in women undergo a sophisticated cycle of growth and apoptosis at every childbirth. Further, the breast tissue regresses at menopause due to declining steroid hormone levels that lead to a loss of glandular tissue and increase in fatty tissue.^31^ The cellular pathways for breast cancer and normal breast development are intertwined. The mammary epithelial cell proliferation during adolescence is induced by primary sex hormones—estrogen and progesterone. They induce physiological changes through adolescence, menarche, menstrual cycle, pregnancy, lactation, cessation of lactation, and menopause.^17^ Estrogen and progesterone exert cellular functions on target organs by association with nuclear and membrane estrogen (ER) and progesterone (PR) receptors.^32^ Estradiol is the predominant estrogen in non‐pregnant females, whereas estrone and estriol dominate during pregnancy and after menopause, respectively.^33^ Estrogen is majorly described as a breast cancer‐promoting hormone.^33^, ^34^ Further, synthetic progesterone exposure correlates with an enhanced breast cancer risk.^35^ In contrast, results of a randomized clinical trial suggest a more beneficial effect of preoperative progesterone intervention independent of the PR expression and menopausal status of women.^36^ These effects are independently corroborated by others.^37^ Moreover, other studies have reported the anti‐mitogenic and anti‐metastatic effects of progesterone through pathways that are independent of the PR status.^38^, ^39^, ^40^

Epidemiological studies have confirmed strong correlation between following reproductive factors and breast cancer risk: (i) early onset menarche, (ii) irregular menstrual cycle, (iii) early pregnancy, (iv) multiple childbirths, (v) never breastfeeding, (vi) late menopause, and (vii) hormone replacement therapy.^41^, ^42^ These phases are typically associated with remarkable hormonal changes. Moreover, the hormonal imbalances, time, and frequency of these changes increase the risk of carcinogenesis in the breast cells. Early onset menarche is associated with high cancer grade, lymph‐node involvement, and poor prognoses.^43^ Prolonged exposure to estrogen and progesterone due to early menarche and late menopause is the primary reason for a high risk of breast cancer in women with no other pathological predisposition.^44^ In contrast, parity (or ≥ 24 weeks of pregnancy) is associated with a 50% decrease in the risk of breast cancer. Finally, hormone replacement therapy (HRT) has been associated with occurrence of breast cancer in postmenopausal women.^45^ Many studies have reported a high association of HRT with an increased risk of breast cancer and poor prognosis. However, the benefits of HRT for relieving severe menopause‐related complications, such as osteoporosis and heart disease, support the necessity of HRT for postmenopausal women.

## 3 GUT MICROBIOME AND CHANGES IN ITS COMPOSITION WITH MENSTRUAL PHASE

The homeostasis and health of humans intimately depends on the microbiota, and dysbiosis leads to diseased state.^8^ The gut microbiota composition is influenced by host genetic factors and environmental conditions, including diet.^46^, ^47^ Thus, the host–gut microbiota interactions control the host activities. An early menarche (age ≤ 11 years) is associated with lower gut microbial diversity and lower abundance of *Firmicutes* members than later menarche (age ≥ 12 years).^48^ The severity of the uncomfortable and stressful gastrointestinal symptoms associated with menstruation, such as dysmenorrhea, abdominal pain, bloating, diarrhea and headache, vary with the menstrual phase each month.^49^ These effects may be aggravated by consumption of oral contraceptives; but probiotics have been shown to alleviate the menstrual discomforts.^50^ For instance, ingestion of *Bifidobacterium* has been shown to reduce gastrointestinal symptoms and abdominal pain.^51^ Thus, microbiota composition and menstrual cycle, similar to other host responses, may have a strong association. The composition of the gut microbiota is known to change significantly during pregnancy, including alterations in diversity and phyla contribution.^52^ Long‐term treatment with conjugated estrogen + bazedoxifene, which is used to alleviate menopause‐related symptoms, affects the gut microbiome composition and estrogen‐metabolizing capacity of the gut.^53^ Nuriel‐Ohayon et al.^54^ identified a significant enrichment of *Bifidobacterium* in pregnant women and female mice than in non‐pregnant women and mice. *Bifidobacterium* levels increased in response to progesterone. Therefore, pregnancy and regular hormonal fluctuations act as strong modulators of the composition of the gut microbiota. Furthermore, postmenopausal women show overall altered composition and a trend toward less diverse gut microbiomes than premenopausal women.^55^ The microbial sulfate transport system is prevalent, whereas microbial β‐glucuronidase is less prevalent, in postmenopausal than in premenopausal women. Microbial β‐glucuronidases are involved in estrogen reactivation and incidence of breast cancer.^56^ These differences are connected with the levels of metabolites of progesterone and estrogen in the serum, indicating a role for postmenopausal gut bacteria in the retention of sex hormones. Moreover, menopause‐related changes in the microbiota in postmenopausal women are linked to worse cardiometabolic profiles.^55^

Postmenopausal women show changes in the overall composition and a decrease in the levels of pathogenic strains of *E. coli* and *Shigella* than premenopausal women. The differences in gut microbiome between post‐ and pre‐menopausal women are equivalent to those between men and postmenopausal women, indicating that alterations in the gut microbiota during menopause could be due to a depletion in levels of female sex hormones.^57^ Thus, postmenopausal blood levels of progesterone and estrogen are influenced by levels of intestinal microbial species and functions relevant to menopause or vice‐versa. Moreover, changes in the gut microbiome composition may increase or decrease the duration of exposure of women to both the hormones.

Peters et al.^55^ reviewed that loss of estrogen and progesterone in postmenopausal women correlates with a reduction in the deconjugating capacity of gut microbiome. Moreover, postmenopausal women have a significantly lower abundance of β‐glucuronidase ortholog than premenopausal women, and this ortholog is linked with multiple menopause‐depleted species. For instance, the levels of *Akkermansia muciniphila* are diminished in postmenopausal women. *A. muciniphila* has been favorably linked to the number of estrobolome orthologs, especially in postmenopausal women. *A. muciniphila* expresses β‐glucuronidase and aryl‐sulfatase, and its levels are favorably linked with levels of progesterone metabolites. Therefore, *A. muciniphila* may be involved in the deconjugation, reactivation, and retention of sex hormones. Additionally, these processes deplete levels of *A. muciniphila* during menopause due to loss of conjugated sex hormone substrates. However, enterohepatic recycling of sex hormones by the gut bacteria may be a significant driver of systemic postmenopausal sex hormone levels as the ovarian hormone synthesis is largely absent in postmenopausal women. Patients with breast cancer have a less diverse gut microbiota, but greater abundance of members of *Clostridiales*.^58^ Moreover, the relative abundances of 45 microbial species are significantly different in the gut of postmenopausal patients with breast cancer than in gut of postmenopausal controls.^59^ Further, sex and gonadectomy have been shown to correlate with the composition of the gut microbiota. For instance, levels of testosterone, dihydroxyprogesterone, and allopregnanolone positively correlate with levels of *Blautia*; whereas, those of testosterone, allopregnanolone, pregnanolone, progesterone, and dihydroxyprogesterone negatively correlate with levels of *Roseburia*.^60^

The administration of oral contraceptives fails to alter the overall composition of gut microbiome in healthy women.^61^ However, dietary changes affect microbial communities in the gut in terms of both composition and function, which may influence innate and adaptive immune systems of the host. Commensals significantly impact the development and responses of the immune system, and thus, they may also affect outcomes of immunological diseases.^62^ Moreover, various autoimmune, allergic, or inflammatory disorders and malignancies have been investigated for the prevention, aggravation, or induction of gut microorganisms.^63^ Thus, an intact microbiome can help in prevention of multiple adverse outcomes in humans.

A healthy infant's gut microbiota and the well‐being of the female host are both influenced by the bacterial community found in the human milk and breast tissue. The bacteria found in the breast tissue may originate from the gut through the skin, nipple, or blood. The gut bacterial translocation theory hypothesizes that the gut microbiota is transferred through the nipple–areolar orifices, nipple–oral contact during lactation, increased intestinal permeability, and/or sexual contact from the skin to the breast tissue.^64^ The most prevalent phylum in the breast tissue is *Proteobacteria*, followed by *Firmicutes*. The bacterial diversity found in the breast is greater than that in vagina, but comparable to that in other sites (such as the gut).^65^ Banerjee et al.^21^, ^66^ identified distinct microbial patterns in patients with triple‐positive and triple‐negative breast cancer; whereas, samples with ER+ and HER2+ status shared similar microbial signatures. Using microarray‐based screening method, they also elucidated presence of specific viromes and microbiomes that are enriched in ER+, but less enriched in triple‐negative, breast cancer samples.^66^ These distinctive or shared characteristics between the breast cancer subtypes provide a new perspective on the functions of the microbiome in breast cancer. A study of the breast microbiota DNA using breast cancer (ER‐positive) and paired normal adjacent tissues identified relative enrichment of *Methylobacterium radiotolerans* and *Sphingomonas yanoikuyae* in the tumor and paired normal tissues, respectively. This inverse correlation between the two species suggests that dysbiosis is linked to the incidence of breast cancer.^22^ Further, a study by Chen et al.^67^ indicated that a healthy breast microbiota could control the growth of opportunistic pathogens, such as *Staphylococcus* and *Corynebacterium*, in the breast of individuals with mastitis. Moreover, their study highlighted the differences in the immune activity in the breast cancer subtypes from patients with Asian and Caucasian origins. Additionally, the composition of the tissue microbiome could be influenced by the gut microbiome and vice‐versa. Taken together, the influence of tissue microbiota, in addition to gut microbiota, on the development of different breast cancer subtypes should be investigated in detail.

## 4 GUT MICROBIOME‐INDUCED CHANGES IN LEVELS OF STEROID HORMONES

**FIGURE 1:**
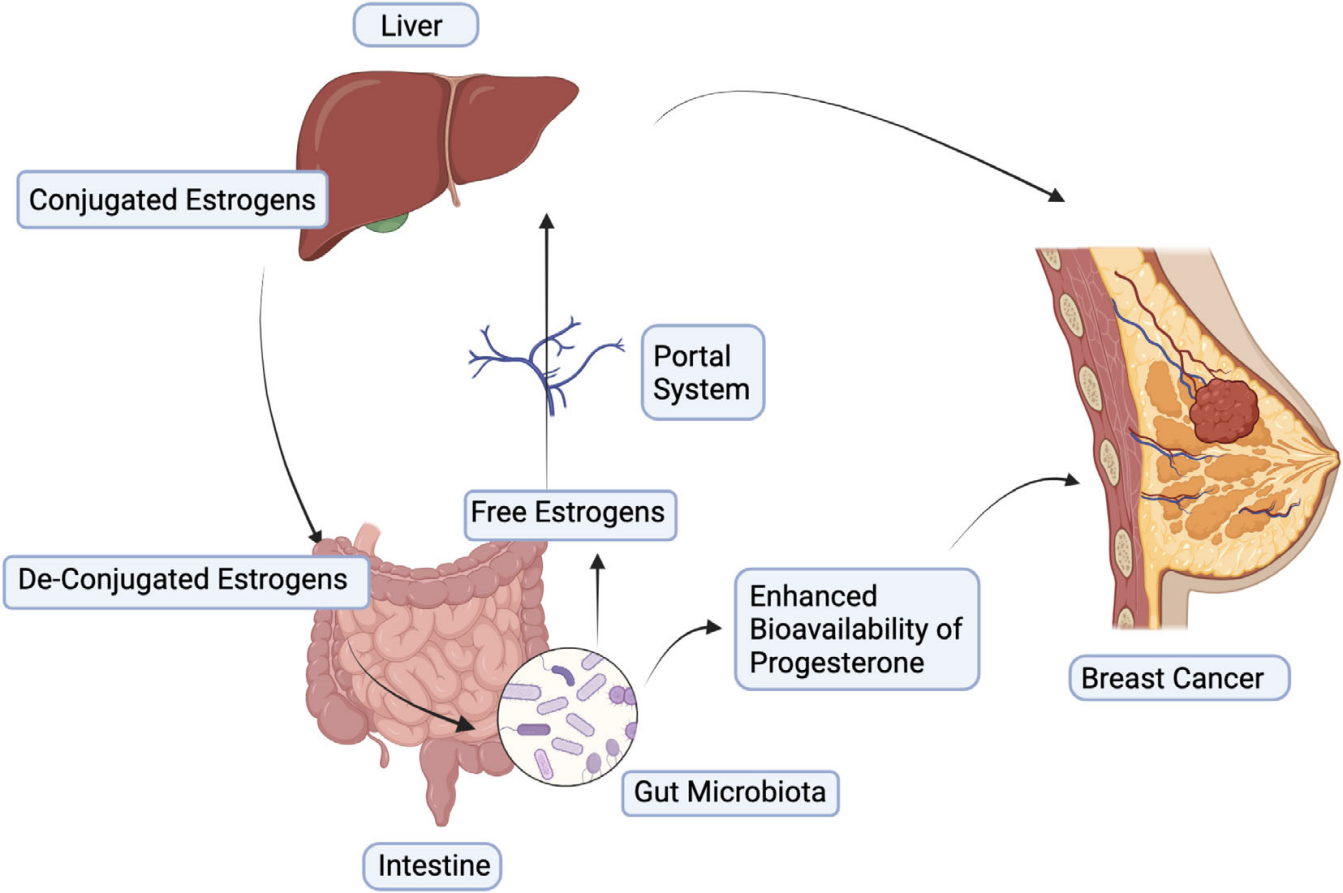
Gut microbiome influences the levels of estrogen and progesterone that modulate the incidence and progression of breast cancer.

Certain human diseases caused by microorganisms show sex hormone‐dependency. For instance, female mice show elevated risk of infection with *Mycobacterium marinum* when they are administered with testosterone. Castrated male mice, on the other hand, show reduced propensity for infection. This suggests that testosterone is the primary reason for increased infectivity of *M. marinum*.^68^ An increased susceptibility of female mice to typhoid‐causing *Salmonella typhimurium* has been linked to estrogen, while progesterone confers resistance to the infection.^69^ Additionally, progesterone has been shown to induce growth of *Lactobacillus* sp. in ovariectomized mice and aids in preventing anxiety‐like behavior and depression.^70^ As the levels and exposure of estrogen and progesterone have been linked with the occurrence of breast cancer, studies have also explored the ability of the gut microbiome to metabolize these hormones. The composition of the gut microbiome is a reflection of different host properties, such as mode of delivery, diet, environmental exposure, genetic makeup, and medications, especially antibiotics. It also favors tumor development and influences response to anticancer therapies, such as chemotherapy and hormonal therapy.^71^ Chronic inflammation, genotoxicity, and perturbation of cellular microenvironment and host metabolism are caused by host–microbe interactions. The development of 16S rRNA sequencing, microarray‐based screening, and metagenomics has allowed evaluation of gut microbiome communities that can metabolize estrogen through the activity of β‐glucuronidases.^28^ Women of reproductive age show dynamic changes in composition of the microbiome in the gut and other body sites, including the vagina and oral cavity, especially during the follicular (estrogenic) and luteal (progestogenic) phases.^72^ The capacity of organisms residing in the human gut to metabolize estrogen and progesterone has been studied since the 1980s—*Bacteroides melaninogenicus* show a high affinity to estrogen and progesterone and can metabolize them.^73^ The intestinal microbiome is capable of metabolizing androgens and estrogens by hydrolytic, reductive, and oxidative reactions.^74^ Further studies have suggested that a majority of gut bacteria have β‐glucuronidase and β‐glucosidase enzymes that can deconjugate estrogens and allow their reabsorption into the bloodstream.^75^ The gut microbiota has the capacity to perform 21‐dehydroxylation or 16*α*‐dehydroxylation of corticosteroids or sex hormones. Moreover, gut microbiota can regulate the levels of testosterone and secretion of androgen by performing 17*β*‐reduction of androgens.^76^ Interestingly, these enzymes are absent in mammalian cells, and thus, highlights the uniqueness of the gut microbial enzymes. Estrobolome is the aggregate of enteric bacterial products that are capable of metabolizing human estrogens.^77^ Conjugated estrogens are eliminated through bile, urine, and feces—approximately 65% of estradiol is recovered in the bile, 10%–15% in the feces, and a sizable proportion is reabsorbed into the bloodstream. The deconjugation performed by the β‐glucuronidase activity of the gut bacteria, such as the *Clostridium leptum* and *Clostridium coccoides* cluster and *Escherichia/Shigella* bacterial group, aids in the reabsorption of hepatically conjugated estrogens that re‐exert their biological functions. Therefore, an estrobolome‐high activity of deconjugating enzymes can encourage the reabsorption of free estrogens, raising estrogen levels and possibly altering the breast tissue. Two genes, *gus* and *BG*, encode β‐glucuronidase in *Firmicutes* and *Bacteroidetes*, respectively. β‐glucosidases are predominantly observed in Gram‐positive *Firmicutes*, *Bacteroides thetaiotaomicron* and *Bifidobacterium* spp.; whereas, β‐glucuronidase activity is observed in some *Firmicutes* within clostridial clusters XIVa and IV.^75^ Further, oral vancomycin has been shown to induce changes in the gut microbiome composition that leads to desulfation of sulfated progesterone, which derails maternal bile acid homeostasis during pregnancy.^78^ Next, several foods, herbs, and spices contain phytoestrogens and phytoprogesterones^79^ that are metabolized by the gut microbiome, and lead to enhanced bioavailability of circulating estrogen and progesterone in women. Prolonged postmenopausal estrogen supplementation has been shown to impact the microbiome composition and enzyme activity, which can affect their ability to metabolize estrogen.^53^ Taken together, as shown in Figure 1, the gut microbiome alters the circulating steroid hormone levels and activity that modulate the incidence of breast cancer, and that more detailed investigations are necessary to better model the associations.

## 5 INFLUENCE OF GUT MICROBIOME COMPOSITION ON INCIDENCE AND PROGRESSION OF BREAST CANCER

Gut microbiome responds to changes in physiological states and is associated with disease occurrence. Perturbations in the composition of the gut microbiome are associated with the development of inflammatory and autoimmune diseases and cancer. Studies suggest a strong association between several cancer types and the microbiome, including breast cancer.^80^ In addition to extrinsic factors, intrinsic factors, such as age, menopausal state, obesity, and ethnicity also play a significant role in shaping the gut microbiota composition.^64^ In general, patients with breast cancer and benign breast lesions show a reduced gut microbiome diversity than healthy individuals. Obesity is known to reduce the diversity of the gut microbiome, and is a risk factor for the incidence and progression of breast cancer.^81^, ^82^, ^83^ For instance, the proportion of *A. municiphila* is decreased and is inversely correlated with body fat in patients with breast cancer. However, administration of probiotics, such as *Bifidobacterium longum* BB536 and *Lactobacillus rhamnosus* HN001, improves the gut bacterial diversity and metabolic functioning.^84^ Thus, whether prolonged administration of such probiotics could improve the gut microbiome composition in patients with breast cancer should be investigated in detail.

Interestingly, microbes residing in organs distant from the breast are also associated with the occurrence of breast cancer through diverse mechanisms. Specifically, the gut microbiome is capable of differentially altering the incidence of steroid hormone receptor‐positive^85^ and ‐negative^86^ breast cancer. The abundance and composition of the gut microbiota have been shown to affect: (i) the concentration of circulating steroid hormones; (ii) interaction of microbiome with the gut–brain axis that has a strong correlation with hormonal regulation; (iii) levels of metabolites of the gut microbiota with tumorigenic or antitumorigenic potential; and (iv) crosstalk of gut microbiome with immune system as it is an important effector of immune response and inflammation.^23^ The varied effects of increase and decrease in the levels of gut microbiome components on the circulating levels of hormones and specific changes in breast cancer are presented in Table 1.

**TABLE 1:** Role and impact of gut microbiome on influencing the effects of hormones and their impact on breast cancer.

Organisms	Influence on hormone levels	Impact on breast cancer	References
*Clostridium leptum* ↑ *Clostridium coccoides* ↑ *Alistipes* ↑ *Citrobacter* ↑ *Dermabacter* ↑ *Marvinbryantia* ↑ *Propionibacterium* ↑ *Tannerella* ↑	Synthesize β‐glucuronidase, β‐glucosidase that increase levels of estrogen	↑ (Activation of procarcinogens or inactivation of anticancer drugs that are conjugated to glucuronide molecules)	^75^, ^85^
*Escherichia/Shigella* ↓ *Aldercreutzia equolifaciens* ↓ *Eggerthella sp* ↓ *Lactobacillus mucosae* ↓	Metabolize phytoestrogen into equol and enteroligans	↓ (Inhibit matrix metalloproteins that abrogate metastasis and increase demethylation of *BRCA1* and *BRCA2*)	^29^
*Firmicutes* ↑ *Collinsella* ↑	Synthesize β‐glucuronidase that increases levels of estrogen	↑ (Activation of procarcinogens or inactivation of anticancer drugs conjugated to glucuronide molecules)	^75^, ^85^
*Bacteroides thetaiotaomicron* ↑	Synthesize β‐ glucosidase that increases levels of estrogen, and phytoprogestins which leads to enhanced bioavailability of circulating progesterone	↑ (Activation of procarcinogens or inactivation of anticancer drugs conjugated to glucuronide molecules)	^75^
*Bifidobacterium* ↑	Synthesize β‐glucosidase that increases levels of estrogen	↑ (Activation of procarcinogens or inactivation of anticancer drugs that are conjugated to glucuronide molecules)	^75^
*Blautia* ↑ *Cetobacterium* ↑ *Eubacterium* ↑ *Hafnia* ↑ *Mitsuokella* ↑ *Turicibacter* ↑ *Weissella* ↑ *Yokenella* ↑	Synthesize β‐glucosidase that increases levels of estrogen	**↑** (Alterations in carbohydrate metabolism, and in ß‐galactosidase activity observed in cancer cells)	^85^

A decrease in levels of butyrate‐producing bacteria reported in postmenopausal women is associated with chronic inflammation and a high risk of breast cancer.^59^ While some gut microorganisms can deconjugate estrogen, others can metabolize phytoestrogen into equol and enterolignans that can protect against breast cancer. Equol is a soy isoflavone metabolite that can be produced by intestinal bacteria, such as *Adlercreutzia equolifaciens*, *Escherichia coli*, *Eggerthella sp*, and *Lactobacillus mucosae*.^29^ S‐equol has a potent estrogenic and antioxidant activity. It can regulate cellular pathways, such as inhibition of matrix metalloproteins that abrogates metastasis and increases demethylation of *BRCA1* and *BRCA2*.^87^ S‐equol has also been reported to alter the activity of breast cancer resistance proteins, cell proliferation, and apoptosis.^88^ Furthermore, free fatty acids (FFA) of varied chain lengths are produced by gut microbiota. The activation of FFA receptor 2 (FFAR2) by short chain fatty acids leads to elevated levels of E‐cadherin in mesenchymal cells that indicates the potential of this metabolite in mesenchymal‐to‐epithelial transition, thereby decreasing metastasis.^58^ FFAR3 activation inhibits invasion by reducing ERK phosphorylation. The anaerobic gut microbiota belonging to *Clostridales* are responsible for production of secondary bile acids, such as lithocholic acid that activates TGR5, leading to inhibition of epithelial‐to‐mesenchymal transition and VEGF pathway. Further, cadaverin, produced by the gut microbiome components such as *Shigella flexneri*, *Shigella sonnei*, *E. coli*, and *Streptococci* group, reduces the aldehyde dehydrogenase 1‐positive cancer stem cell population in the 4 T1 murine breast cancer cells.^89^ Finally, lithocholic acid, a gut microbial product, can reduce the incidence of early breast cancer by decreasing cancer cell proliferation, expression of vascular endothelial growth factor, and metastasis. It can also enhance antitumor immune response and oxidative phosphorylation and Krebs cycle.^90^ Therefore, the capacity of the gut microbiome to metabolize components of diet and produce factors that stimulate host responses significantly influences the breast function and cancer incidence.

Banerjee et al.^66^ conducted a microarray‐based study of microbial signatures of breast cancer types using a pan‐pathogen strategy. Patients with ER‐positive cancer showed the highest, whereas those with triple‐negative breast cancer (TNBC) showed the lowest, gut microbiome diversity. TNBC samples formed a distinct cluster due to abundance of *Aggregatibacter* and presence of *Plagiorchis* and *Trichostrongylus*. ER‐positive and triple‐positive subtypes showed a microbial profile that is intermediate of the two. Analysis of patients with TNBC showed that a higher abundance of *Bacillus*, *Mucor*, *Nodaviridae*, *Toxocara*, and *Trichophyton* significantly correlated with a prolonged disease‐free survival. Further, Dieleman et al.^91^ compared the microbiome of different breast cancer types with that of the normal breast tissue. They reported that luminal A tumors exhibit the highest abundance of *Xanthomonadales* than luminal B tumors that show the highest abundance of *Clostridium*. *Methylobacterium* is less abundant in a majority of the hormone‐positive breast cancer tissues than in healthy breast tissue. In HER2+ cancer samples, *Akkermansia* is abundant while *Streptococcaceae* and *Ruminococcus* are abundant in TNBC samples. Additionally, ethnicity is known to have a profound effect on the gut microbiome, along with differences in the types and prevalence of breast cancer. Smith et al.^92^ attempted to study differences in the breast microbiota in non‐Hispanic black and non‐Hispanic white women. *Ralstonia* is prevalent in non‐Hispanic black women with breast cancer, whereas *Xanthomonadaceae* is prevalent in non‐Hispanic white women with breast cancer. Moreover, the population of *Bacteroidetes* is also diminished in non‐Hispanic black women than that in non‐Hispanic white women. Moreover, *Streptococcaceae* are enriched in patients with triple‐negative breast cancer, along with a stage‐dependent enrichment of *Bosea*. Taken together, the gut and breast microbiome influence the incidence and progression of breast cancer in an intricate manner. Additional ethnicity‐based studies are warranted to better appreciate the effects of environmental conditions and genetic makeup on the role of gut microbiome in breast cancer.

## 6 INFLUENCE OF GUT MICROBIOME COMPOSITION ON TREATMENT OUTCOMES IN WOMEN WITH BREAST CANCER

The host's gut and the microbiota have a symbiotic interaction that helps maintain homeostasis, and aids in digestion, metabolism, and immunological responses. A study on preclinical mouse model suggested that administration of probiotics fails to improve the microbiota recovery back to baseline after treatment with antibiotics. Instead, autologous fecal microbiota transplantation significantly improves recovery within days.^93^ This highlights the importance of the presence and absence of particular microbes in the gut. The composition of the gut microbiome also influences response to therapeutic agents, including chemotherapy, anti‐HER2 therapy, immunotherapy, and hormonal therapy.^94^ Terrisse et al. investigated the association between gut microbiome and response of patients with breast cancer to adjuvant chemotherapy. They showed that specific commensals from the gut microbiome influence breast cancer prognosis in patients and aggressiveness in mice model, and treatment with chemotherapy alters the balance of favorable and unfavorable gut microbial species.^9^ A recent study by Vernaci et al. suggested that patients with TNBC that respond to anthracycline‐based chemotherapy have a higher α‐diversity and higher species richness than patients that relapse.^95^ Further, Bawaneh et al. showed that administration of doxorubicin alters the composition of the gut microbiome, and this or high‐fat diet‐derived fecal microbiota transplantation reduces the chemotherapeutic efficacy of doxorubicin.^86^ Moreover, the anticancer drugs alter the composition of the gut microbiome in mice, and germ‐free mice or those pretreated with antibiotics show resistance to anticancer agents, such cyclophosphamide.^96^ In addition, the gut microbiota alters host response to immunotherapy. An intact commensal microbiota modulates myeloid‐derived cell functions in the tumor microenvironment, thus ensuring optimal responses to cancer therapy.^97^ Furthermore, Vétizou et al. showed that the antitumor, immunotherapeutic effect of CTLA‐4‐specific 9D9 antibodies is modulated by *Bacteroides* sp. in mouse model and patients with metastatic melanoma.^98^ Routy et al. observed that an abnormal gut microbiome is the primary factor leading to resistance to immune checkpoint inhibitors targeting epithelial tumors. They also showed that oral administration of *Akkermansia municiphila* improved the anti‐PD‐1 blockade therapy in original non‐responders.^99^ An absence of immunostimulatory microbiota and overabundance of immunosuppressive microbiota is being considered as the primary cause of treatment failures.^100^ A study by Wu et al. indicated that PR/ER status, tumor grade and stage, parity and body mass index failed to associate with alpha diversity and phyla differences. However, they showed that patients with HER2‐positive tumors showed a lower alpha diversity, lower abundance of *Firmicutes* and higher abundance of *Bacteriodetes* than that in patients with HER2‐negative tumors.^48^ Another study on preclinical mice model and patients with HER2‐positive tumors indicated that the gut microbiome plays an essential role in the response to trastuzumab.^101^ Taken together, gut microbes modulate the anticancer and immune responses in a subtype‐specific manner.

Enzymes produced by the gut microbiota can metabolize more than 271 orally‐administered drugs, including sulfasalazine, lovastatin, omeprazole, and risperidone. The enzymes can either activate, inactivate, or toxify the drugs ingested by humans.^102^ For instance, *Clostridium scindens* (ATCC 35704) can desmolytically metabolize dexamethasone, prednisone, prednisolone, cortisone, and cortisol.^103^ Furthermore, a study by Iida et al.^97^ highlighted the necessity of an intact gut microbiota for anticancer efficacy of oxaliplatin and cisplatin in mice. Interestingly, presence of microbes with the capacity to metabolize estrogen and inhibitors of estrogen signaling (e.g., tamoxifen) in the gut can influence the outcomes of ER‐positive breast cancers. Specifically, the utility of *gus*‐inhibitors for the treatment of ER‐positive breast cancers is currently being tested.^104^ Thus, these studies highlight the importance of a healthy and enriched gut microbiota for the patients to respond to therapy. Finally, maternal obesity influences the responsiveness of daughter to anticancer and immunomodulating treatment.^82^ However, dietary habits that promote the growth of microbiome capable of synthesizing short‐chain fatty acids may aid in reversing the daughter's resistance to breast cancer therapy. Therefore, the mechanisms and microbial pathways associated with the development and progression of breast cancer should be studied in more detail to provide better treatment options for patients with breast cancer, especially as the steroid hormone levels and the breast local microenvironments differ with menopausal status. As endogenous and preoperative progesterone intervention influence the outcomes of breast cancer,^27^, ^36^ it would be interesting to elucidate whether the composition of the gut microbiome alters the beneficiary effects of progesterone in the patients. Additionally, studies should focus on elucidating the long‐term effects of gut dysbiosis in postmenopausal women receiving hormone replacement therapy, especially on the occurrence of breast cancer. An ethnicity‐based approach, as microbiome composition differs based on environmental conditions, may help identify answers to such hypotheses and reduce the burden of breast cancer. Finally, the role of phages in controlling the levels and activity of pathogenic microbes needs to be thoroughly investigated as they may serve as a systematic approach for preventing dysbiosis.^105^

## 7 CONCLUSION

This review describes the intricate mechanisms by which the gut microbiome regulates the levels of estrogen and progesterone by increasing their deconjugation and bioavailability, and thus, influences the incidence and therapy response of breast cancer. It has also discussed different reproductive factors and specific microbial species that could be targeted to prevent breast cancer and improve disease outcomes. The study emphasizes the necessity to maintain a diverse and healthy gut microbiome for ensuring appropriate response to anticancer agents to improve the treatment outcomes and survival. Finally, the review also highlights the need for more detailed investigations on the role of gut microbiome in progression of breast cancer.

## AUTHOR CONTRIBUTIONS

**Shilpa S. Chapadgaonkar:** Formal analysis (equal); investigation (equal); writing – original draft (equal). **Srashti S. Bajpai:** Formal analysis (equal); investigation (equal); visualization (equal); writing – original draft (equal). **Mukul S. Godbole:** Conceptualization (lead); formal analysis (equal); investigation (equal); supervision (lead); visualization (equal); writing – original draft (equal); writing – review and editing (lead).

## FUNDING INFORMATION

This study did not receive funding support.

## CONFLICT OF INTEREST STATEMENT

The authors have stated explicitly that there are no conflicts of interest in connection with this article.

## ETHICS STATEMENT

Not applicable.

## Data Availability

Data sharing not applicable to this article as no datasets were generated or analyzed during the current study.
